# Horses’ (*Equus Caballus*) Laterality, Stress Hormones, and Task Related Behavior in Innovative Problem-Solving

**DOI:** 10.3390/ani9050265

**Published:** 2019-05-22

**Authors:** Laureen Esch, Caroline Wöhr, Michael Erhard, Konstanze Krüger

**Affiliations:** 1 Department of Veterinary Sciences, Faculty of Veterinary Medicine, Animal Hygiene and Animal Husbandry, Chair of Animal Welfare, Ethology, Ludwig Maximilian University Munich, Veterinaerstr 13/R, 80539 Munich, Germany; caroline.woehr@tierhyg.vetmed.uni-muenchen.de (C.W.); m.erhard@tierhyg.vetmed.uni-muenchen.de (M.E.); 2 Department Equine Economics, Economics and Management, Faculty Agriculture, Nuertingen-Geislingen University, Neckarsteige 6-10, 72622 Nuertingen, Germany; konstanze.Krueger@hfwu.de; 3 Zoology/Evolutionary Biology, University of Regensburg, Universitaetsstraße 31, 93053 Regensburg, Germany

**Keywords:** innovative behavior, brain lateralization, glucocorticoid metabolites, behavioral traits, equine cognition

## Abstract

**Simple Summary:**

In order to ensure species-appropriate horse keeping and management, knowledge about horses’ cognitive abilities is essential. One parameter used to measure the cognitive abilities of a species is their capacity for innovative behavior. In this study 16 horses where confronted with a novel problem, i.e., an unknown feeder. When a horse emptied the feeder completely it was considered to be innovative. We found 25% of the horses to be innovative. Horses’ propensity to innovate was mediated by individual behavioral differences and former life experiences. We conclude that horses’ keeping conditions and welfare may be improved by environmental enrichment which promotes the development of innovative behavior.

**Abstract:**

Domesticated horses are constantly confronted with novel tasks. A recent study on anecdotal data indicates that some are innovative in dealing with such tasks. However, innovative behavior in horses has not previously been investigated under experimental conditions. In this study, we investigated whether 16 horses found an innovative solution when confronted with a novel feeder. Moreover, we investigated whether innovative behavior in horses may be affected by individual aspects such as: age, sex, size, motor and sensory laterality, fecal stress hormone concentrations (GCMs), and task-related behavior. Our study revealed evidence for 25% of the horses being capable of innovative problem solving for operating a novel feeder. Innovative horses of the present study were active, tenacious, and may be considered to have a higher inhibitory control, which was revealed by their task related behavior. Furthermore, they appeared to be emotional, reflected by high baseline GCM concentrations and a left sensory and motor laterality. These findings may contribute to the understanding of horses’ cognitive capacities to deal with their environment and calls for enriched environments in sports and leisure horse management.

## 1. Introduction

The growing importance of horses as sports and leisure partners [1] and the demand for improved animal welfare [2] calls for a better understanding of equine cognition to ensure that their mental performance is neither under- nor overestimated [3]. It also calls for improving horse management and training programs [1]. One of the potential measures for animal cognitive abilities is considered to be innovativeness [4,5,6,7], which is often measured by using a problem-solving task [8,9,10,11]. Innovativeness is defined as a novel solution to environmental challenges and animals finding solutions to a novel problem can be considered to be innovative [12]. Previous studies investigated whether and how animals develop an innovative solution when confronted with a novel problem (e.g., [13,14,15,16,17,18]) such as an unfamiliar feeder (different avian species: [19]; primates: [12,20]).

Innovative problem-solving abilities can be influenced by several factors. Firstly, the age, sex, or size may be influential [8]. In a variety of species young animals display more innovative behavior than adults (primates: [12]; hyenas: [21]; birds: [22]; meerkats: [11]). However, in a study on anecdotal reports about innovative behavior in primates, adults showed higher innovation rates than juveniles [23]. Furthermore, the ability for innovative problem-solving may differ between the sexes (great tits: [24]; primates: [23]; meerkats: [11]). The individual size of the animal, motor diversity, as well as body strength may also influence the individuals’ abilities to solve the problem as the animals have to deal with an experimental setup installed at a certain height [8].

Secondly, the propensity to behave innovatively in a problem-solving task may be influenced by the degree of the animal’s lateralization, as described for New Caledonian crows [25] and parrots [26]. Sensory laterality is defined as the preferential use of sensory organs of a particular side and motor laterality is defined as the preference for the use of forelimbs, paws, claws, or hands of a particular side [27]. In horses, the left forelimbs and their laterally placed eyes and ears are largely connected to the right brain hemisphere, and vice versa [28]. While the left hemisphere has been shown to be preferentially used for established learned responses, categorization of stimuli and approach behavior, the right hemisphere has been proposed as being dominant in information processing for emergency reactions, stress responses, novelty, and social interactions [29,30,31,32,33,34,35,36]. The motor laterality is assumed to be the result of brain hemispheric modulation through former experiences and training [27,37]. Sensory laterality may be independent of previous experiences and may change more flexible between left and right organ use with different tasks than reported for motor laterality [31,38]. Rogers [39] suggests that more strongly lateralized animals should have enhanced cognitive abilities and, should be more adaptive to novel challenges, contradicted by Marshall–Pescini [40], who found, that, strongly right- and left-pawed dogs, were slower to obtain feed from a novel feeding device.

Thirdly, stress hormone concentrations may influence an animal’s learning performance as low or moderate levels of glucocorticoids may enhance cognitive function, while high glucocorticoid concentrations may reduce the learning performance [41,42,43]. Individual glucocorticoid concentrations can be the result of former life experience [44] and affect how an animal reacts when trying to find an innovative solution for a novel problem [8].

Fourthly, the animal’s success in an innovative problem-solving task may also depend on the animal’s activity, motivation, and persistency, as it is shown for common mynas [18], guppies [45], great tits [24], and chimpanzees [46]. The attention needed to solve a problem may be reduced in highly active animals, as in common mynas [18]. This is contradictory to great tits, where success in an innovation task, may reflect higher activity levels [24]. The latency to approach a novel problem [8] is used as a measurement to test the animal’s motivation for problem-solving [13,18,19]. In addition, the tenacity to continue a behavior, even when it is not rewarded [47], and the persistency of interactions with a novel problem [8] have been considered to be important for problem-solving, as it offers a greater diversity of approaches and increases the chance for finding a solution [8]. Innovative problem-solving behavior, such as operating an unfamiliar feeder, may be performed by chance first and then reinforced by the acquisition of food rewards [24]. Therefore, horses’ feeding motivation [48] may be crucial for their propensity to innovate in a feeding task.

A recent study based on anecdotal reports showed that horses are innovative in opening door and gate mechanisms (Krueger et al. submitted, [49]), but experimental evidence for innovativeness in horses is still missing. The aim of this study was to establish whether 16 horses of mixed ages and breeds would show innovative behavior when confronted with an unfamiliar feeder. We asked whether innovative behavior of horses would be affected by their a: age, sex, and size [50,51,52,53], b: motor and sensory laterality [35], c: glucocorticoid concentrations [54], and d: trait related behavior—latency, activity, tenacity, persistency, and individual food motivation [8,47,48,55].

## 2. Materials and Methods

### 2.1. Study Period and Location

The tests were conducted from May to June 2017 in a private riding stable in central Bavaria, Germany. The test horses were housed in individual boxes with straw bedding. Each horse received individually portioned concentrated feed and hay three times per day according to its needs and had free access to water and a mineral licking stone. Each day, the horses were walked for one hour in a horse walker, turned out on a single paddock for one to three hours without feed or grass, returned to the stable for feeding, and afterwards turned out on pasture with social contact for one to three hours. In addition, the horses were ridden, driven, and/or worked on the ground at leisure level. On the experimental days, the tested horses were exercised and handled as usual to preserve the daily routine.

### 2.2. Animals

The study started with 17 horses of mixed ages, sex (8 mares, 9 geldings) and breeds (13 warmbloods and 4 ponies [56,57]). All horses were in good feeding condition (BCS 5–6 on a scale from 1 to 9 [58]). None of the horses had any previous experience with the feeder.

Prior to the experiment, the horses were tested to see whether they would eat the feed to be used in the experiment. Each horse was offered the same amount of feed (150 g) and the time taken to consume it was documented. One warmblood horse did not accept the feed and was excluded from the experiment. Hence the study included 16 horses (8 geldings and 8 mares; age 7–25 years, median 15 years). Furthermore, all horses were subjected to a thorough physical examination by a veterinarian before the start of the test. This included an examination of the eyes and a check of the teeth and the mobility of the head. None of the horses had any physical restrictions.

### 2.3. Experimental Area

**Figure 1:**
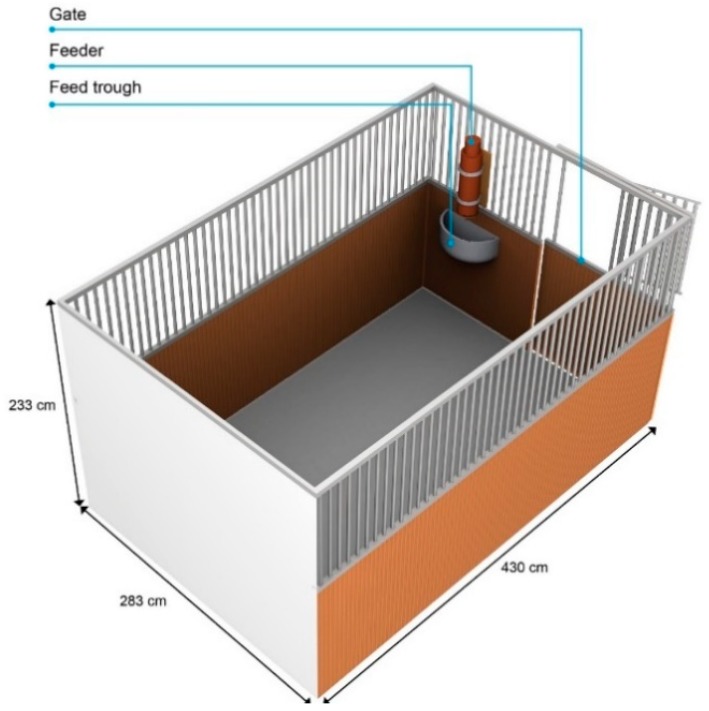
The horse’s box as experimental area, type A. See Supplementary Material Figure S1 and S2 for type B.

The horses were tested in their own boxes, and each box conformed to the measurements prescribed in the German guidelines for horse keeping [59]. The boxes were closed on four sides and were made of wood up to a height of 130 cm and of lattice bars up to the height of 233 cm. The feeder was installed above the feeding trough. The feeding troughs were either on the left or the right side of the box (Figure 1) and the entrance to the box was beside the feeding trough.

### 2.4. Feeder and Testing Conditions

**Figure 2:**
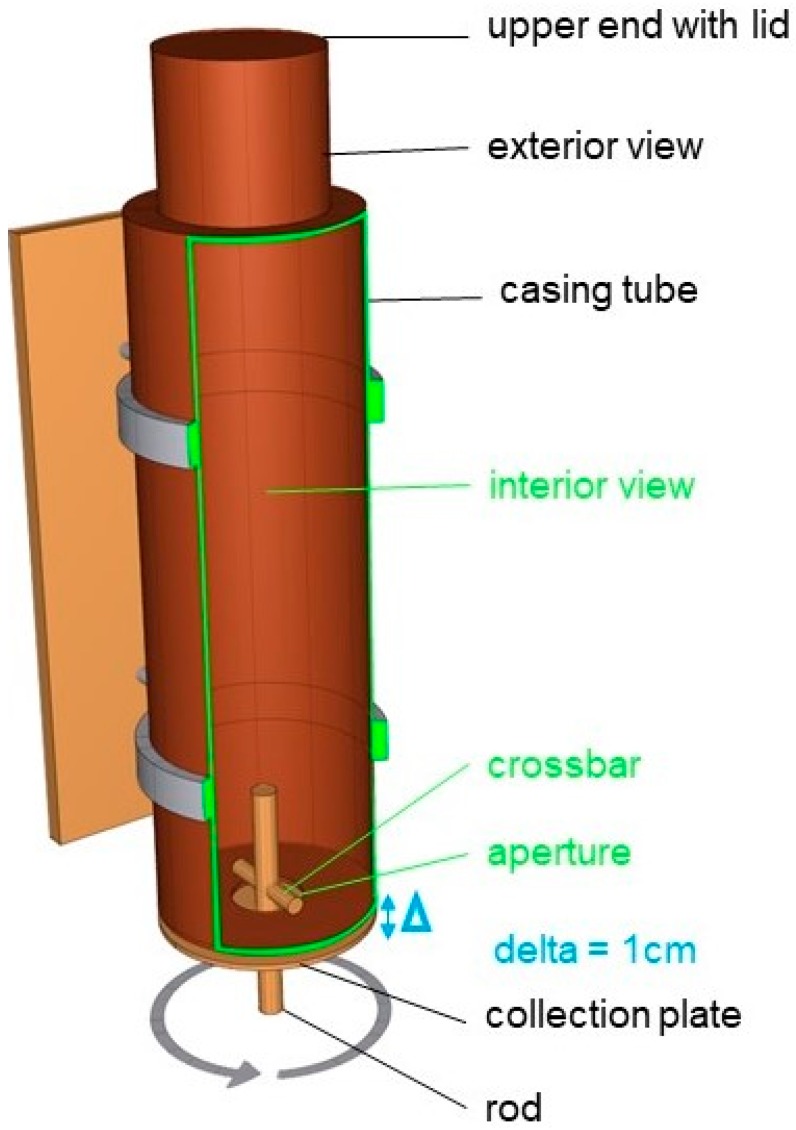
Operation mode of the feeder. When the animals turn the rod, feed falls through the aperture (Ø 6.5 cm) on a plate 1 cm below. Further turning of the rod and the plate makes the feed fall into the horses feeding trough. The rod can be turned to the right and left side.

The feeder was similar to a casing tube with a pendulum (Figure 2 and Figure S3). When a horse moved the rod with the muzzle, a crossbar was rotated inside the feeder, and small quantities of feed trickled onto the collection plate, and with further rotation of the rod the feed fell from the collection plate in the feeding trough. The feeder was filled with three kg (according to the feeding recommendation of the feed producer DERBY) concentrate pellets (DERBY Struktur-Fit: 9.09 MJ DE; 21.7% Crude fiber; 12.5% crude protein). The upper end of the tube was closed with a lid which could be removed for refilling (see Figure S3). Inside the horse’s box, the feeder was fastened with tension belts on the grid above the feeding trough.

### 2.5. Experimenters

Two experimenters participated in the study: Experimenter 1 installed the experimental set up, checked and filled the feeder, collected the data, and analyzed the videos and observed the horses. For calculating the interrater reliability of the horse’s sensory and motor laterality the experimenter 1 was supported by a trainee. Experimenter 2 was a member of the stable staff and led the horses to and from the boxes. After releasing the test horse at the entrance to the box, he/she immediately turned away from the horse and the box and moved out of sight of the horse. The stable staff consisted of three men and one woman.

### 2.6. Experimental Procedure

The testing of each horse took place over 38 h in total. This time span was chosen because horses needed 28 h at most to empty the feeder in a preliminary experiment on testing the function of the feeder. The preliminary experiment was conducted in another stable, with additional 27 horses of mixed ages (1–32 years, median 13.85 years), sex (19 mares, 6 geldings, 2 stallions) and breeds (12 Arabian horses, 7 quarter horses, 2 warmblood horses, 6 crossbreed horses). Over 38 h, the horse had the opportunity to learn to operate the feeder. The experimental duration was set at 38 h in total, due to stable management and for adding a time buffer. The feeder was filled with food to provide positive reinforcement if the horse was successful. To exclude the possibility of social information transfer between test horses a screen was installed between the test horses’ boxes and the neighboring boxes. Camcorders were used to record the experiment (see Section 2.8).

### 2.7. Habituation Phase

The test horses were habituated and tested one by one. On the day before the test, the screen was installed between the test horse’s box and the neighboring horse. The test horse then had time to habituate to the screen. A horse was habituated when it stood relaxed in the box and showed usual activities such as eating [60,61]. All horses habituated to the screen in <5 min.

### 2.8. Test Phase

On the test day, the feeder was installed in the box while the test horse was on the pasture. The tube of the feeder was filled with three kg of feed. Camcorders were installed, the picture quality was checked, and the recording was started. We continuously collected data with two camcorders (Raspberry Pi 2 CamBox, Pollin, Pfoerring, Germany) using motion detectors so that only the horse’s movements were recorded. One camera focused on the feeder, the other provided an overview of the box. The cameras were synchronized for recording analog test dates and times. An infrared lamp was installed in the box for night recording. The videos were stored via a router (FRITZ! Box 3170, AVM, Berlin, Germany) on a notebook (NC10 Plus, Samsung, Seoul, South Korea).

The test horses were collected at pasture and led to their box. Since the horses were used to being handled from the left, the test horses were always led at their left side until they reached the entrance to their box. They were released in front of the box and they walked into their box alone to prevent the experimenter from influencing the horse’s first exploration of the feeder. The experiment started as soon as the horse entered the box with its entire body. The next day, experimenter 1 checked how much feed was left in the tube. If the horse had emptied it completely, the test was completed and the feeder was removed. If there was still feed in the tube, the test continued for another day and stopped after 38 h.

### 2.9. Data Collection

First, we determined the propensity to behave innovatively. The amount of consumed feed was weighed after the test and considered a measurement of innovativeness. When the feeder was completely empty the horse was successful and termed an ‘innovative problem-solver’. Learning the mechanism of the feeder was considered to be established in these horses, as emptying the feeder included multiple action sequences at different locations, the feeder and the food trough. If a horse just ate a limited amount of the feed but did not empty the whole feeder within the two days, we could not prove established, repeatable learning processes [7,8] and could not rule out that some may have opened the feeder simply by chance without any understanding of the mechanism. We considered the horse partially successful and termed them as a ‘by chance problem-solver’. In the case of a horse not being able to get feed from the feeder it was defined as ‘non-problem-solver’. The factors that may influence the propensity to innovate have been determined as follows.

#### 2.9.1. Laterality

The sensory laterality of the horses was analyzed from the video recordings by noting with which side of the head the horse turned towards the novel feeder for their first approach, when contacting the feeder. This was visible in all cases either from the camera which recorded the feeder or from the camera which recorded the whole box. An approach of the horse to the feeder was counted, when a contact with the feeder followed the approach. A lateral approach was counted when the horse twisted its head and body more than 10° to the left or to the right to approach and contact the feeder (Supplementary Material Figures S4 and S5) with a particular eye. 10° shifts between right and left eye use were clearly visible and indicate monocular eye choice, even though, the binocular filed comprises 35° in horses [62,63]. When the horse approached the feeder head-on, the approach was rated neutral, i.e., binocular (Supplementary Material Figures S4 and S5).

A new approach and contact was counted when the interaction with the feeder was interrupted for more than 10 seconds, in accordance with the duration of short-term memory reported in horses [64,65]. Sensory laterality was determined on the basis of the number of occurrences within the time period of the test (cf. [66]). 5% of the videos were rated by a second person to evaluate interobserver reliability in rating the sensory laterality of the approaches. The agreement with experimenter 1 was Kappa = 0.88.

The motor laterality of the test horses was analyzed while they were grazing on the pasture in the weeks before the experiment was started. We documented which front leg was placed in front every 10 seconds until we had 200 recordings. These observations were distributed over three consecutive days for each horse [35]. To determine the interobserver reliability the motor laterality counts of three horses were observed by a second person on the pasture and Kappa between the two observers was calculated (K = 0.96).

A sensory and motor laterality index [27,30] was calculated for each horse by applying the formula LI = (R − L)/(R + L). Where L stands for number of left scores and R for the number of right scores. The index can take a value between −1 and 1, in which the negative score stands for a preference for the left sensory organs or legs, while a positive result greater than 0 up to +1 indicates a preference for the right ones and an index equal 0 means an ambilateral use of the left and ride side. The absolute value of the lateral index (|LI|) is a measurement of the strength of lateral bias irrespective of the direction of the bias. To determine whether a preference of an individual horse was significant, Z scores (L − (L + R/2)/√ ((L + R)/4) were calculated [27]. A z-score ≥ 1.96 or ≤ −1.96 indicates a lateral bias, a value between these two scores indicates no lateral bias (ambilateral).

#### 2.9.2. Stress

We evaluated the horses GCMs as a measure of the individual’s base level of stress [67]. The mean glucocorticoid baseline value was determined from three fecal samples per horses taken on three consecutive days prior to the experiment between 6:00 and 7:00 a.m., as the glucocorticoid values underlie circadian variations [67]. Another sample was taken at 6:30 a.m. on the second day of each individual’s test phase and provided the stress hormone concentration of the first test day because of the 24 h excretion delay of horse feces [68,69]. To preserve the samples, the feces were dried as described and published elsewhere [70]. According to Krueger et al. [70] 1.5–2 g of fresh feces (from five different areas in the pile) were weighed with a spoon scale (technoline KW-120), rolled in a teabag (Cilia S) and dried with 20 mL silica gel (SiO_2_; Steiner Chemie ST Trockenperlen) in a 50 mL tube.

For GCM extractions from dried samples, we used 0.5 g of horse feces. Cortisol metabolites were extracted from horse feces with the simplified method described by Flauger et al. [71]. In short: 0.1 g dry feces, plus 1 mL water and 4 mL methanol were vortexed for 30 min. The methanolic suspension was centrifuged. A small part of the supernatant was diluted in assay buffer and frozen until EIA analysis. The glucocorticoid values are given in ng/g. The samples were analyzed at Nuertingen-Geislingen University by using enzyme immunoassays validated for the analysis of GCMs in horse feces.

#### 2.9.3. Task Related Behavior

We primarily used the camera recordings which focused on the feeder for the analysis. The recordings from the overview camera were used for clarification when a video sequence was ambiguous. All times are given in minutes.

(a) Activity

As the horses’ movements started the video cameras, the sum of the duration of the video sequences is equal to the sum of being in motion of the horse and was used to document the horse’s activity.

(b) Persistency

When the feeder was touched with the horse’s muzzle a contact was registered. The number of each test horse’s contact with the feeder is defined as the horse’s persistency to solve the problem.

(c) Latency

The real time duration until first contact was taken as a measurement of the individual latency to approach the feeder.

(d) Food motivation

The time taken until the feed was consumed in the pre-trial was used as a measure for possible motivational influences of the feed on the success in the experiment. The longer a horse needed to eat the feed, the less it may have been motivated to operate the feeder [48].

(e) Tenacity

The duration of time spent with the feeder was calculated by summing the durations of contact with the feeder. Thereafter, we calculated the duration each particular horse spent with the feeder in relation to its activity, by dividing the duration spent with the feeder by the horses’ activity. In this way, we could exclude the influence of the individual activity on the horses’ tenacity.

### 2.10. Statistical Analyses

We applied the R-project statistical environment (R version i368 4. 4 2018, R Foundation for Statistical Computing, Vienna, Austria) and the package R-commander. As some variables were not normally distributed (Shapiro–Wilk test), we applied non-parametric tests. We applied generalized linear mixed models (GLMMs) for considering random and fixed effects on the dependent variable ‘amount of food consumed (g)’, as a measurement for whether horses were innovative in a few trials. The model was set at family ‘gaussian’, the ID of the horses considered as random factor and the specific factors age, sex, size, motor and sensory laterality, basal and test value of the glucocorticoid metabolites, activity, latency, persistency, food motivation, and tenacity as fixed factors. After the stepwise removal of factors, the model with the best fit (with the lowest AIC index) was chosen. The complete and reduced models are provided in the Supplementary Material as File S2. For evaluating the significance of the horses’ individual sensory and motor laterality, we calculated z-scores [27] (Supplementary Material Tables S1 and S2). All tests were two-tailed and the significance level was set at *p* < 0.05.

### 2.11. Ethical Statement

We obtained permission from all persons that participated in this study to publish the data. This study was non-invasive, needed no approval by local ethics committees, and complies with the Guidelines for Ethical Treatment of Animals in Applied Animal Behaviour and Welfare Research (ISAE Ethics Committee, 2017).

## 3. Results

We found that 4 horses (25%) out of 16 were innovative problem-solvers and emptied the feeder completely. An additional six horses (37.5%) solved the mechanism of the feeder by chance (by chance problem-solvers) but consumed only some feed (feed consumption: *n* = 6, median = 560 g, min = 350 g, max = 2100 g). The remaining six horses did not manage to get feed from the feeder and were termed non-problem-solvers. The horses’ age and the GCM values of the test day did not influence the innovative problem-solving ability and could be excluded, while reducing the AIC index. Furthermore, the persistency, the motor and sensory laterality, the food motivation (as a measure for the time needed to consume 150 g of feed in a pretest), and the horses’ size (wither’s height) had no significant influence on whether horses solved the problem innovatively (GLMM: *n* = 16, all *p* > 0.01).

### 3.1. Horses’ Sex and Innovative Problem-Solving

The sex tended to influence the propensity to behave innovatively: the innovative problem-solvers were two females and two males, but four of the six horses who solved the problem by chance were males (GLMM: *n* = 16, SE = 0.03, t = −2.809, *p* = 0.07).

### 3.2. Laterality and Innovative Problem-Solving

Of the 16 test horses, 10 showed left motor laterality, 2 right motor laterality, and 4 showed no significant preference of a particular side (ambilateral) (z-scores, Supplementary Material Table S1). Seven horses showed significant left sensory laterality, two showed right sensory laterality and seven were ambilateral (z-scores, Supplementary Material Table S2). Although the motor and sensory laterality had no significant influence on the propensity to behave innovatively, all the four innovative problem-solvers, displayed significant left motor (z-scores, Supplementary Material Table S1) and three of them significant left sensory laterality (z-scores, Supplementary Material Table S2).

### 3.3. Stress Hormones and Innovative Problem-Solving

**Figure 3:**
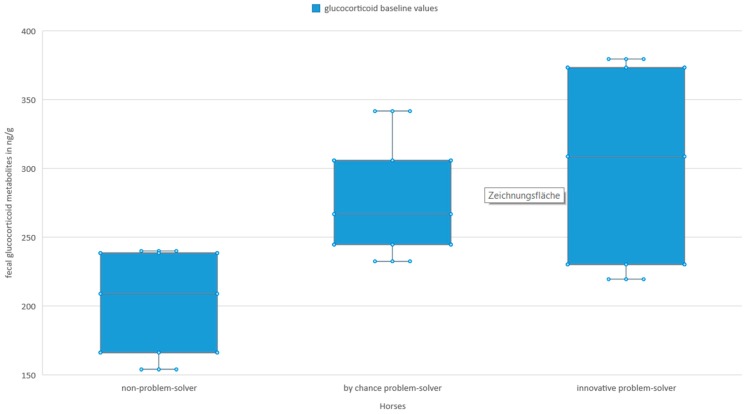
Glucocorticoid metabolites baseline values in the feces of the test horses (innovative problem-solver: *n* = 4, median = 308. 57 ng/g, min = 219.37 ng/g, max = 379.34 ng/g; by chance problem-solver: *n* = 6; median = 266.68 ng/g, min = 232.39 ng/g, max = 341.50 ng/g; non-problem-solver: *n* = 6, median = 208.76 ng/g, min = 153.85 ng/g, max = 239.87 ng/g). The box comprises 50% and the lower and upper whisker 25% of the variability each. The line in the middle visualizes the median.

The GCM baseline values tended to have an effect on the amount of feed consumed (GLMM: *n* = 16, t = 2. 404, SE = 1.85, *p* < 0.095). When comparing groups, on average, innovative problem-solvers and by chance problem-solvers tended to have higher GCM baseline values than the non-solvers (Figure 3).

### 3.4. Task Related Behavior and Innovative Problem-Solving

**Figure 4:**
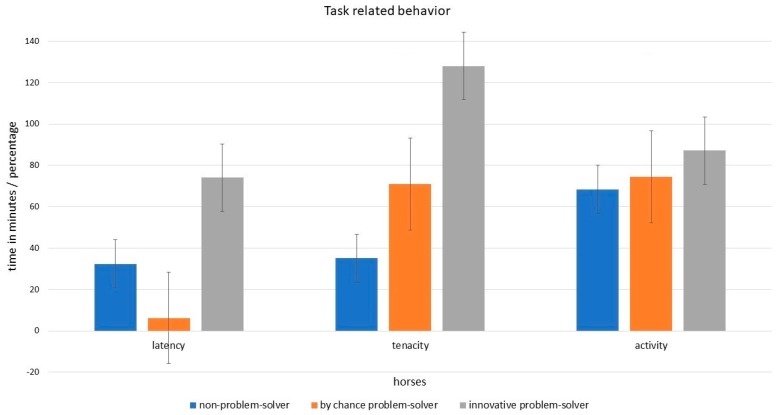
Task related behavior of the test horses. The activity is calculated from the animal’s level of being in motion. As the activity covers a longer lapse of time, the minutes were divided by 10 to be graphically comparable to the other behaviors in this figure. The animals’ latency is calculated from the duration until first contact with the feeder. The duration of time spent with the feeder is the ratio from the time spent with the feeder to the active time. Therefore, the time effort is given as a percentage. The bars indicating the standard errors.

The amount of feed consumed was significantly affected by the horses’ level of activity (GLMM: *n* = 16, t = 3.847, SE = −0.67, *p* = 0.03). When comparing groups, innovative and by chance problem-solvers were more active than the non-problem-solver horses (Figure 4). Furthermore, the time from the beginning of the test until the first contact to the feeder affected the amount of feed consumed (GLMM: *n* = 16, t = 7.141, SE = −0.74, *p* < 0.01). In comparison to the by chance problem-solvers and the non-problem-solvers, the innovative problem-solvers needed significantly more time from the beginning of the test until they contacted the feeder for the first time (Figure 4). The time spent interacting with the feeder had a significant effect on the amount of feed consumed (GLMM: *n* = 16, t = 7.714, SE = 0.14, *p* < 0.00). When comparing groups, on average, horses who solved the problem innovatively and by chance invested significantly more time interacting with the feeder than the non-problem-solvers (Figure 4).

## 4. Discussion

The present study found 25% of 16 horses to be innovative in solving a problem (i.e., in operating an unknown feeder), which is comparable to ratios of innovations in wild vervet monkeys, *Chlorocebus pygerythrus* (32% of 53) [72].

Our findings suggest that problem-solving abilities in horses are mediated by individual differences in task related behavior as shown in previous studies (hyaenas, *Crocuta Crocuta*: [21]; indian myna, *Acridotheres tristis*: [15]; wild meerkats, *Suricata suricatta*: [11]). We found that innovative horses were more active while being tested, which is supported by a study in which horses with higher activity performed better in an acquisition task, than horses with lower activity [55]. In accordance with previous studies [11,17,47], the innovative and by chance problem-solver horses had a higher tenacity to solve the problem, than non-problem-solver horses. This is also supported by studies [73,74] which demonstrated that animals that explore more slowly had higher problem-solving abilities. However, a greater tenacity for problem-solving may prevent success if individuals cannot inhibit making errors [8]. Since errors could have been impeded by blocking an impulsive response, successful performance may also be due to the individuals’ enhanced capacities for inhibitory control [75,76]. As innovative problem-solver horses displayed a greater latency to contact the feeder for the first time than the non-problem-solver horses, we suppose that horses succeeding in an innovative problem-solving task may have an improved inhibitory control. Supported by studies on new caledonian crows [77] and on horses [1], which found that animals displayed more accurate answers when they took more time to understand the concept of a learning task.

In contrast to former studies [8], we could not find a significant influence of the horses’ persistency on their problem-solving abilities, which may be caused by differences in data collection. Previously, persistency was defined as the number of new approaches [8], while in our study, repeated contacts with the feeder within one approach increased the horses’ tenacity and not its persistency. We suppose that a higher tenacity results in a greater variety of trials and an increased number of successful manipulations, as it is claimed for hyaenas [21] and birds [15,78]. However, our findings call for further studies on a larger number of animals to investigate whether differences in task-related behavior may be the key to understanding innovative problem-solving in horses.

The innovative and by chance problem-solver horses of our study had elevated GCM baseline values, which may be the result of repeated stimulations in enriched environments. Hence, the individuals’ life history may affect the individuals’ problem-solving abilities [79]. Individuals’ early life experience may also explain the motor left side bias of the innovative problem solvers and sensory left side bias of three-quarters of the innovative problem-solver horses. Early experiences may have affected the development of hemispheric specialization and resulted in an emotional, right hemispheric (i.e., left motor and sensory sided) cognitive bias [80]. This is supported by the finding that left-handed marmosets showed higher concentrations of the stress hormone cortisol than right-handed marmosets [34].

In summary, the innovative and by chance problem-solvers’ elevated baseline GCM concentrations and the innovative problem-solvers’ preference for the left eye and forelimb may indicate that innovativeness in horses is associated with emotionality [66]. The quality of the emotionality may be positive or negative, as there is evidence for horses preferring sensory organs of the left side for the intake of positive and negative emotional information [29]. The feeder, a food-related novel object, may provoke either positive or negative emotional reactions and the motor and sensory left lateralized innovative horses may have had more brain power available [39,81] for solving this innovation task.

The tendency of geldings to be more innovative may be explained by differences in behavior and learning capacities, as male horses exhibit more play behavior [82] and are considered to learn more quickly, than mares [83]. A follow up study is needed to clarify whether sex is decisive for innovative behavior in horses. Also, the finding that the individual food motivation does not influence the innovative problem-solving ability, indicates that the successful solution of a novel problem may be related to play behavior and may be rewarding enough [84], as it is claimed for rhesus monkeys [85] and cattle [86].

The horses’ size did not hamper their success in operating the novel feeder [8]. Also, the horses’ age had no influence on whether they were innovative in the present study. However, the missing age effect may have been hampered by the fact that the test horses of the present study were rather old (median 15 years) and age differences to younger horses, which proved to learn faster [51,52] and to be more interested in novel stimuli [50] were not evident.

Our findings indicate that innovative horses solved the problem partially through trial and error [18] and positive reinforcement with food motivated them to manipulate the feeder longer [87]. Otherwise a higher tenacity in innovative problem-solving may indicate that individuals’ motivation is goal directed [8]. Furthermore, the fact that innovative horses took more time to approach the feeder may have been a result of their higher inhibitory control or may suggest that some horses solved the problem through reasoning, and therefore through higher cognitive abilities [88,89,90]. However, our first approach to innovative learning in horses did not aim to evaluate learning mechanisms. This needs to be investigated in detail by further studies.

## 5. Conclusions

Our study revealed evidence for horses being capable of innovative problem-solving in operating a novel feeder. Innovative horses of the present study were active, tenacious, and may be considered to have a higher inhibitory control, which was revealed by their task related behavior. Furthermore, they appeared to be emotional, reflected by high baseline GCM concentrations and a left sensory and motor bias. To conclude, we consider that innovative problem-solving abilities in horses may be mediated by inherent behavioral differences and former experiences in the individuals’ life. Environmental enrichment through improved keeping conditions may contribute to the mental welfare in horses.

## Supplementary Materials

The following are available online at https://www.mdpi.com/2076-2615/9/5/265/s1, Figure S1: Perspective view of the horse’s box, type B; Figure S2: Interior view of the horses’ box, type B; Figure S3: Construction of the feeder; Figure S4: Evaluation method of the horses’ sensory laterality; Figure S5: Horse lateral approach with (a) the left eye, (b) the right eye or (c) in a neutral position; Table S1: Ratings of the horses’ motor laterality and z-scores; Table S2: Ratings of the horses’ sensory laterality and z-scores; Figure S6: Motor and sensory laterality of the test horses; File S2: Complete and reduced Generalized linear mixed models.
