# Supplementary material for: Horses’ (Equus Caballus) Laterality, Stress Hormones, and Task Related Behavior in Innovative Problem-Solving
Source: Animals (Basel). 2019 May 22;9(5):265. doi: 10.3390/ani9050265 (PMC6562608; doi:10.3390/ani9050265)
Supplement: Supplementary file 1 [file animals-09-00265-s001.pdf]

animals

Supplementary Material

The horses' (*Equus caballus*) laterality, stress hormones and task related behavior in innovative problem-solving

Laureen Esch [1,2,*], Caroline Wöhr [1], Michael Erhard [1], Konstanze Krüger [2,3]

1. Ludwig Maximilian University Munich, Veterinarian Medicine, Department of animal welfare, ethology, animal hygiene and animal husbandry, Veterinaerstr. 13/R, 80539 Munich, Germany
2. Nuertingen-Geislingen University, Faculty Agriculture, Economics and Management, Department Equine Economics, Neckarsteige 6-10, 72622 Nuertingen, Germany
3. University of Regensburg, Zoology / Evolutionary Biology, Universitaetsstraße 31, 93053 Regensburg, Germany

* Correspondence: laureen.esch@gmx.de; Tel.: 0049 8456 9186099

Figure S1. Overview of the experimental area, the horse's box, type B. The horses were tested in their own boxes. The boxes were closed on four sides and were made of wood up to a height of 130 cm and of lattice bars up to the height of 233 cm.

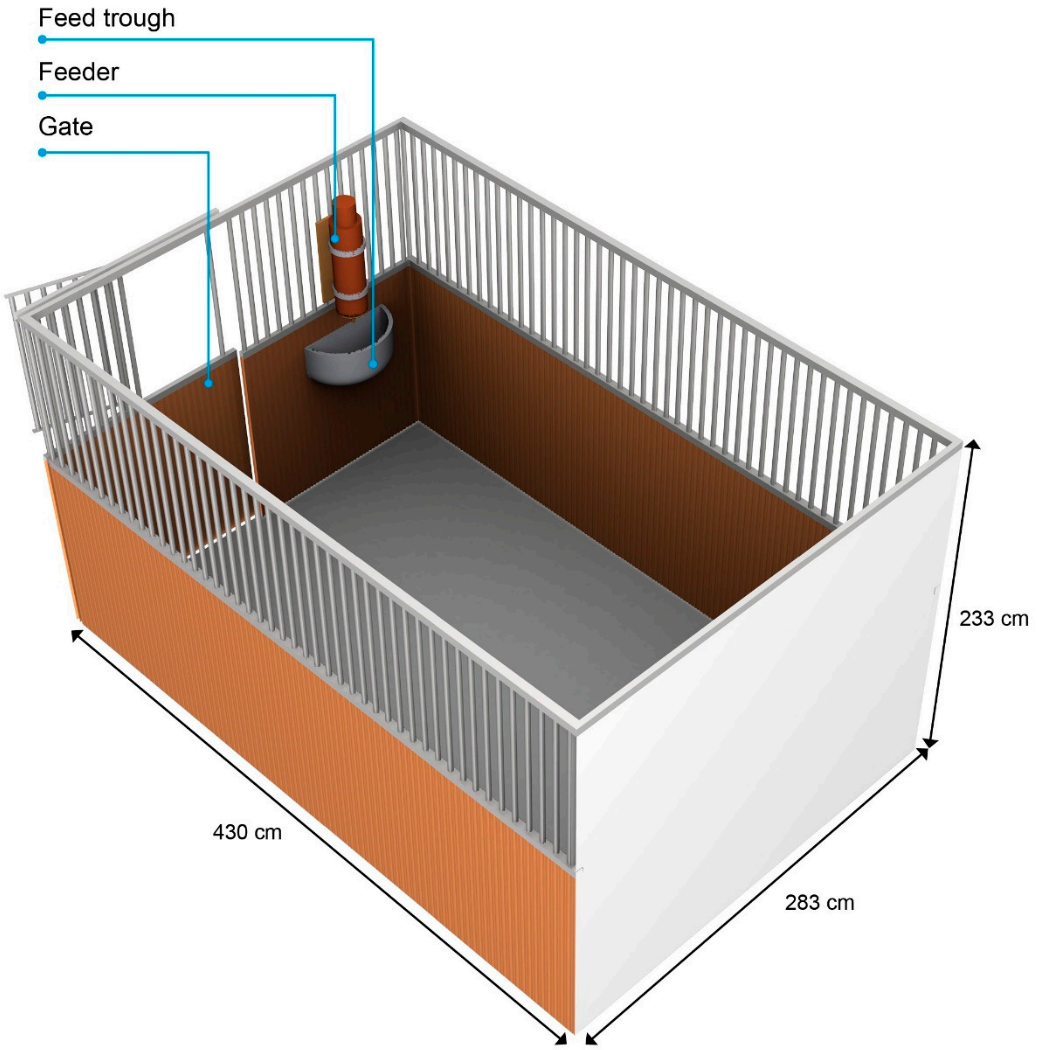

Figure S2. Inside the test area, the horses' box, type B. The feeder was installed above the feeding trough. The feeding troughs were either on the left or the right side of the box and the entrance to the box was on the opposite side from the feeding trough.

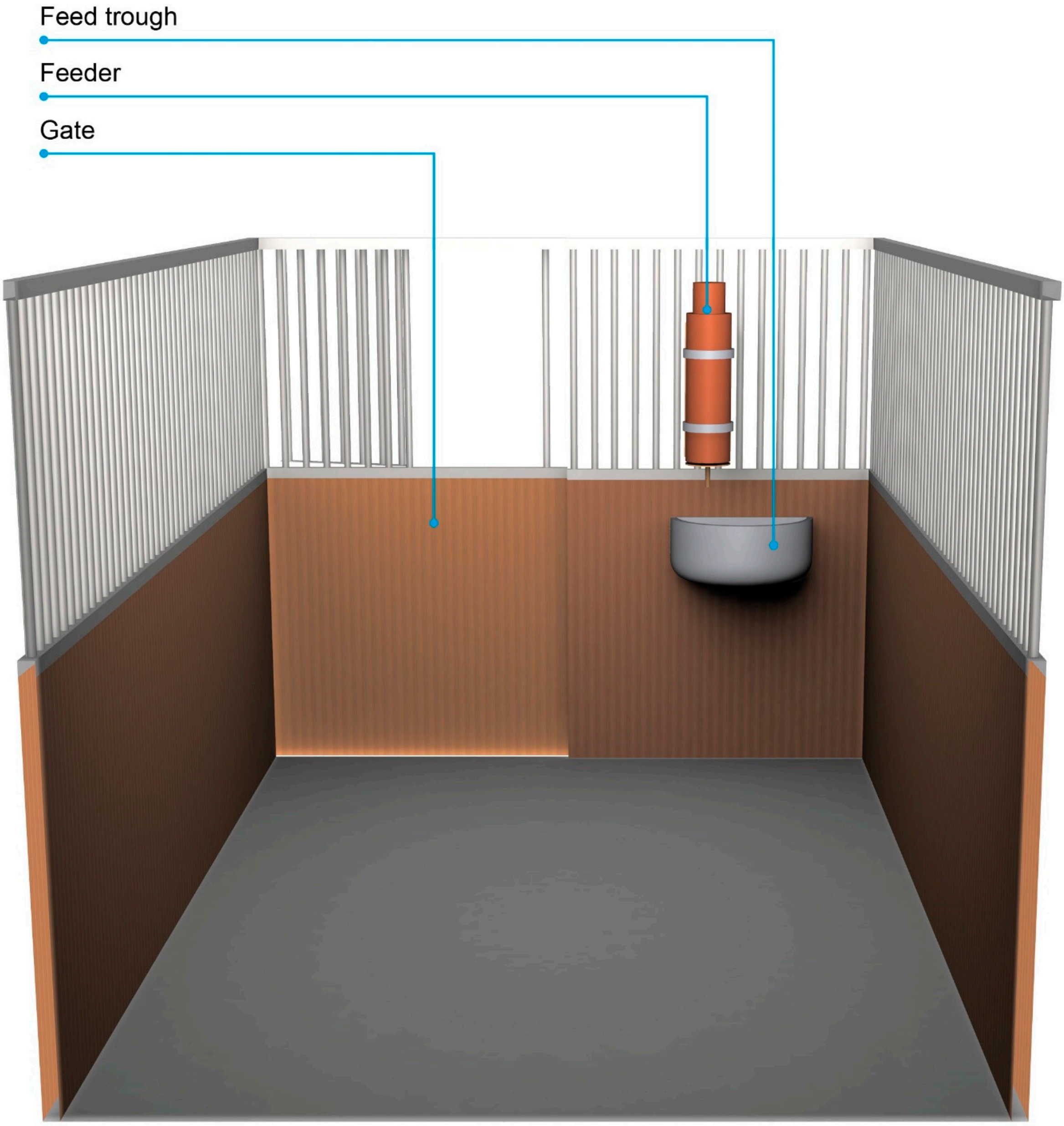

Figure S3. Construction of the feeder. The feeder was similar to a casing tube with a pendulum. When a horse moved the rod with the muzzle, a crossbar was rotated inside the feeder, and small quantities of feed trickled onto the collection plate, and with further rotation of the rod the feed fell from the collection plate in the feeding trough.

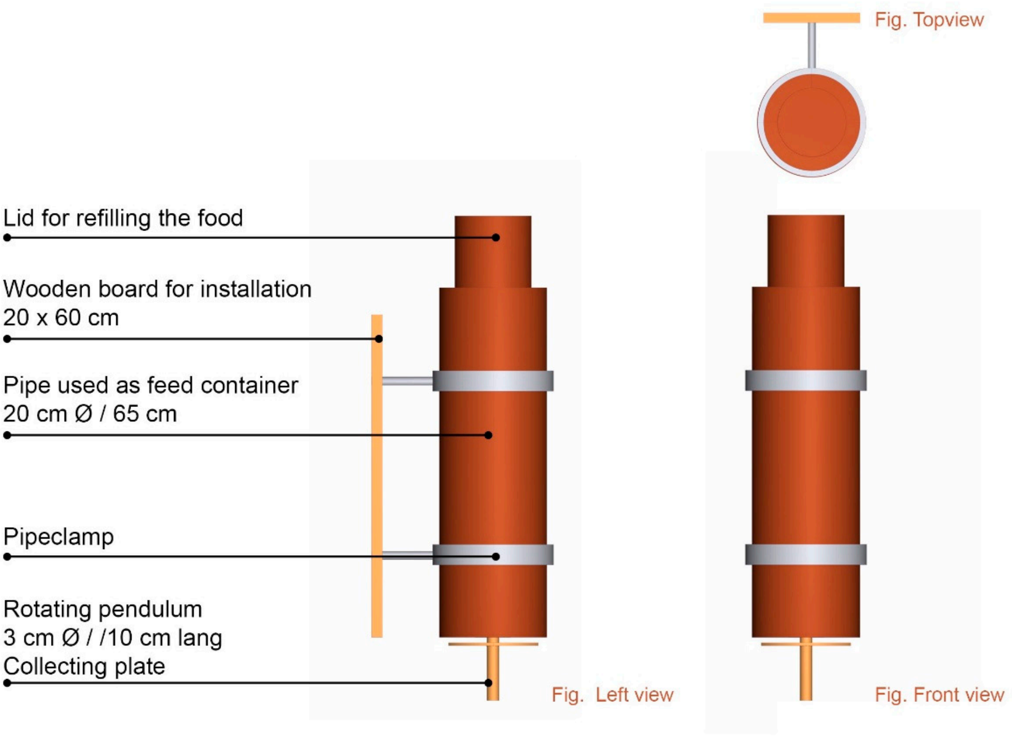

Figure S4. Criteria for evaluating the horses' sensory laterality. An approach of the horse to the feeder was counted when a contact with the feeder followed the approach. A lateral approach was counted when the horse twisted its head or body more than 10° to the left or to the right to approach and contact the feeder

Figure S5. Horse lateral approach with a) the left eye, b) the right eye or c) in a neutral position. The sensory laterality of the horses was analyzed from the video recordings by noting the side of the head the horse turned towards the novel feeder for their first approach, when contacting the feeder.

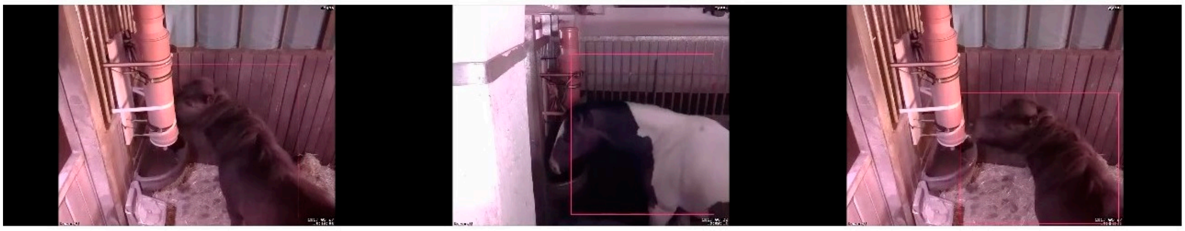

Table S1. Horse motor laterality data. The motor laterality of the test horses was analysed while they were grazing on the pasture in the weeks before the experiment was started. The front leg that was placed forward was recorded every ten seconds until we had 200 recordings. These observations were distributed over three consecutive days for each horse. A sensory and motor laterality index (Austin rogers 2012, 2014) was calculated for each horse by applying the formula $LI = (R - L) / (R + L)$. Where L stands for number of left scores and R for the number of right scores. The index assigns a value between -1 and 1, in which the range -1 to less than 0 stands for a preference for the left sensory organs or legs, while a result between greater than 0 and +1 indicates a preference for the right ones and a result equal to 0 means an ambilateral use of the right and left side. The absolute value of the laterality index ($|LI|$) is a measurement of the strength of lateral bias irrespective of the direction of the bias. To determine whether a preference of an individual horse was significant Z scores $(L - (L + R/2)/ \sqrt{((L + R)/4)}$ were calculated. A z-score ≥ 1.96 or ≤ -1.96 indicates a lateral bias, a value between these two scores indicates no lateral bias (ambilateral). Values of % left bias, calculated as $L/(L + R) \times 100$, are also stated as a

descriptive statistic to give an indication of the degree of bias but these were not used in any of the calculations.

We categorized the horses to the following groups: innovative problem-solvers (ips), by chance problem-solvers (cps) and non-problem-solvers (nps).

**Horse**	**group**	**left**	**right**	**index**	**% left bias**	z-score	laterality group
Jana	cps	137	63	-0,37	68,50	5,23	left
Mirella	nps	197	3	-0,97	98,50	13,72	left
Kalinka	ips	146	54	-0,46	73,00	6,51	left
Floh	nps	94	106	0,06	47,00	-0,85	ambilateral
Melody	nps	78	112	0,18	41,05	-2,47	right
Filou	nps	94	106	0,06	47,00	-0,85	ambilateral
Tessa	nps	173	27	-0,73	86,50	10,32	left
Rimma	cps	121	79	-0,21	60,50	2,97	left
Sam	cps	89	111	0,11	44,50	-1,56	ambilateral
Szölo	cps	184	16	-0,84	92,00	11,88	left
Filly	nps	6	194	0,94	3,00	-13,29	right
Eloisa	ips	182	18	-0,82	91,00	11,60	left
Rio	cps	91	109	0,09	54,50	-1,27	ambilateral
Casuro	cps	184	16	-0,84	92,00	11,88	left
River	ips	179	21	-0,79	89.50	11,17	left
Quiso	ips	136	64	-0,36	68,00	5,09	left

Table S2. Horse sensory laterality data**.** A new approach and contact were counted when the interaction with the feeder was interrupted for more than ten seconds. Sensory laterality was determined on the basis of the number of occurrences within the time period of the test. A sensory and motor laterality index was calculated for each horse by applying the formula LI = (R − L) / (R + L). Where L stands for number of left scores and R for the number of right scores. The index assigns a value between -1 and 1, in which the range -1 to less than 0 stands for a preference for the left sensory organs or legs, while a result between greater than 0 and +1 indicates a preference for the right ones and a result equal to 0 means an ambilateral use of the right and left side. The absolute value of the laterality index (| LI |) is a measurement of the strength of lateral bias irrespective of the direction of the bias. To determine whether a preference of an individual horse was significant Z scores (L − (L + R/2)/ √ ((L + R)/4) were calculated. A z-score ≥ 1.96 or ≤ −1.96 indicates a lateral bias, a value between these two scores indicates no lateral bias (ambilateral). Values of % left bias, calculated as L/(L + R) × 100, are also stated as a descriptive statistic to give an indication of the degree of bias but

these were not used in any of the calculations. We categorized the horses to the following groups: innovative problem-solvers (ips), by chance problem-solvers (cps) and non-problem-solvers (nps).

Horse	group	left	right	index	% left bias	z-score	laterality group
Jana	cps	15	44	0,49	25,42	-3,78	right
Mirella	nps	16	26	0,24	38,10	-1,54	ambilateral
Kalinka	ips	66	6	-0,83	91,67	7,07	left
Melody	nps	5	9	0,29	35,71	-1,07	ambilateral
Floh	nps	78	135	0,27	36,62	-3,91	right
Filou	nps	18	1	-0,89	94,74	3,90	left
Tessa	nps	8	7	-0,07	53,33	0,26	ambilateral
Rimma	cps	10	6	-0,25	62,50	1,00	ambilateral
Sam	cps	20	5	-0,60	80,00	3,00	left
Szölo	cps	22	10	-0,38	68,75	2,12	left
Filly	nps	17	10	-0,26	62,96	1,35	ambilateral
Eloisa	ips	6	5	-0,10	54,55	0,30	ambilateral
Rio	cps	14	2	-0,75	87,50	3,00	left
River	ips	65	12	-0,69	84,42	6,04	left
Casuro	cps	19	12	-0,23	61,29	1,26	ambilateral
Quiso	ips	59	20	-0,50	74,68	4,39	left

File S2. Complete and reduced generalized linear mixed models (GLMMs). We applied generalized linear mixed models for considering random and fixed effects on the dependent variable 'amount of food consumed', as a measurement for whether horses were innovative in a few trials. The model was set at family 'gaussian', the ID of the horses considered as random factor and the specific factors age, sex, size, motor and sensory laterality, basal and test value of the glucocorticoid metabolites, activity, latency, persistency, food motivation and tenacity as fixed factors.

Consumption: amount of consumed feed from the feeder; given in gram

Activity: sum of the duration of the video sequences is equal to the sum of the horses' being in motion, e.g. active; time is given in minutes
Age: given in years
B.ng.g: basal value (mean of 3 consecutive days) of the glucocorticoid metabolites in ng/g
Food motivation: time needed to consume the feed in the pretrial, time is given in minutes
Latency: time duration until first contact is the latency, time is given in minutes
Motor laterality: laterality index
Persistency: number of each test horse's contact with the feeder is the persistency
Sensory laterality: laterality index
Through position: Type B = right (1) or Type A = left (2)
Sex: mare (1) or gelding (2)
Size: measured at wither's height, given in meters
T.ng.g: test value of the glucocorticoid metabolites in ng/g
Tenacity: time spent with the feeder in relation to the agility, given as a percentage

Including all factors

```
Call:
glm(formula = consumption ~ (activity + age + B.ng.g + food.motivation + latency
+ motor.laterality + persistency + (sensory.laterality/through.position) + sex +
size + T.ng.g + tenacity)/ID, family = gaussian(identity),data = Dataset)

Deviance Residuals:
       1         2         3         4         5         6         7         8
  86.145  -151.377     1.265    58.570   -27.156    33.607   192.137    86.371
       9        10        11        12        13        14        15        16
-197.804    -5.734  -112.043  -161.069   130.767     7.362   -80.303   139.262

Coefficients:

                                                   Estimate
(Intercept)                                       3.445e+02
activity                                          3.003e+00
age                                               4.314e+01
B.ng.g                                            2.165e+00
food.motivation                                  -1.352e+02
latency                                           5.935e+00
motor.laterality                                 -5.550e+02
persistency                                      -6.661e+00
sensory.laterality                               -2.455e+03
sex                                              -3.413e+02
size                                             -2.628e+03
T.ng.g                                            8.679e-01
```

```
tenacity                                                    1.243e+02
sensory.laterality:through.position                         1.870e+03
activity:age:B.ng.g:food.motivation:latency:motor.laterality:persistency:sensory.
laterality:through.position:sex:size:T.ng.g:tenacity:ID  -1.392e-13

                                                            Std. Error
(Intercept)                                                 7.135e+03
activity                                                    2.730e+00
age                                                         1.533e+02
B.ng.g                                                      1.214e+01
food.motivation                                             1.418e+03
latency                                                     3.125e+00
motor.laterality                                            8.272e+02
persistency                                                 7.686e+00
sensory.laterality                                          4.931e+03
sex                                                         1.638e+03
size                                                        6.094e+03
T.ng.g                                                      4.866e+00
tenacity                                                    6.230e+01
sensory.laterality:through.position                         3.845e+03
activity:age:B.ng.g:food.motivation:latency:motor.laterality:persistency:sensory.
laterality:through.position:sex:size:T.ng.g:tenacity:ID   1.970e-13

                                                            t value
(Intercept)                                                 0.048
activity                                                    1.100
age                                                         0.281
B.ng.g                                                      0.178
food.motivation                                            -0.095
latency                                                     1.899
motor.laterality                                           -0.671
persistency                                                -0.867
sensory.laterality                                         -0.498
sex                                                        -0.208
size                                                       -0.431
T.ng.g                                                      0.178
tenacity                                                    1.996
sensory.laterality:through.position                         0.486
activity:age:B.ng.g:food.motivation:latency:motor.laterality:persistency:sensory.
laterality:through.position:sex:size:T.ng.g:tenacity:ID  -0.707

                                                            Pr(>|t|)
(Intercept)                                                 0.969
activity                                                    0.470
```

```
age                                                        0.825
B.ng.g                                                     0.888
food.motivation                                            0.939
latency                                                    0.309
motor.laterality                                           0.624
persistency                                                0.545
sensory.laterality                                         0.706
sex                                                        0.869
size                                                       0.741
T.ng.g                                                     0.888
tenacity                                                   0.296
sensory.laterality:through.position                        0.712
activity:age:B.ng.g:food.motivation:latency:motor.laterality:persistency:sensory.
laterality:through.position:sex:size:T.ng.g:tenacity:ID   0.608

(Dispersion parameter for gaussian family taken to be 200664.3)
Null deviance: 24443000  on 15  degrees of freedom
Residual deviance:   200664  on  1  degrees of freedom
AIC: 228.39

Number of Fisher Scoring iterations: 2
```

After stepwise removal of factors:

```
Call:
glm(formula = consumption ~ (activity + B.ng.g + food.motivation +
    latency + motor.laterality + persistency + (sensory.laterality/through.positi
on) +
    sex + size + tenacity)/ID, family = gaussian(identity), data = Dataset)

Deviance Residuals:
       1          2          3          4          5          6          7          8
  85.680   -163.420    -12.157     38.217    -15.463     13.607    137.214    136.568
       9         10         11         12         13         14         15         16
-195.507      6.973    -87.439   -134.663    109.683     12.632    -27.953     96.028

Coefficients:

                                                 Estimate
(Intercept)                                    -1.842e+03
activity                                        2.575e+00
B.ng.g                                          4.435e+00
food.motivation                                 3.121e+02
latency                                         5.292e+00
motor.laterality                               -4.819e+02
```

```
persistency                                        -5.817e+00
sensory.laterality                                 -1.346e+03
sex                                                -8.359e+02
size                                               -8.364e+02
tenacity                                            1.099e+02
sensory.laterality:through.position                 8.907e+02
activity:B.ng.g:food.motivation:latency:motor.laterality:persistency:sensory.late
rality:through.position:sex:size:tenacity:ID  -9.967e-10

                                                    Std. Error
(Intercept)                                         1.186e+03
activity                                            6.692e-01
B.ng.g                                              1.845e+00
food.motivation                                     2.027e+02
latency                                             7.412e-01
motor.laterality                                    2.336e+02
persistency                                         3.368e+00
sensory.laterality                                  5.941e+02
sex                                                 2.976e+02
size                                                8.018e+02
tenacity                                            1.425e+01
sensory.laterality:through.position                 3.610e+02
activity:B.ng.g:food.motivation:latency:motor.laterality:persistency:sensory.late
rality:through.position:sex:size:tenacity:ID   2.675e-10

                                                    t value
(Intercept)                                        -1.554
activity                                            3.847
B.ng.g                                              2.404
food.motivation                                     1.539
latency                                             7.141
motor.laterality                                   -2.063
persistency                                         -1.727
sensory.laterality                                  -2.265
sex                                                 -2.809
size                                                -1.043
tenacity                                             7.714
sensory.laterality:through.position                  2.467
activity:B.ng.g:food.motivation:latency:motor.laterality:persistency:sensory.late
rality:through.position:sex:size:tenacity:ID   -3.726

                                                     Pr(>|t|)
(Intercept)                                          0.21808
activity                                             0.03099*
```

```
B.ng.g                                          0.09556’
food.motivation                                 0.22139
latency                                         0.00565**
motor.laterality                                0.13115
persistency                                     0.18261
sensory.laterality                              0.10841
sex                                             0.06734’
size                                            0.37355
tenacity                                        0.00453**
sensory.laterality:through.position             0.09027’
activity:B.ng.g:food.motivation:latency:motor.laterality:persistency:sensory.late
rality:through.position:sex:size:tenacity:ID    0.03368*

Signif. codes:  0 '***' 0.001 '**' 0.01 '*' 0.05 '.' 0.1 ' ' 1

(Dispersion parameter for gaussian family taken to be 53267.29)
Null deviance: 24443000  on 15  degrees of freedom
Residual deviance:   159802  on  3  degrees of freedom
AIC: 220.75

Number of Fisher Scoring iterations: 2
```
